# The Specification and Maturation of Nociceptive Neurons from Human Embryonic Stem Cells

**DOI:** 10.1038/srep16821

**Published:** 2015-11-19

**Authors:** Erin M. Boisvert, Sandra J. Engle, Shawn E. Hallowell, Ping Liu, Zhao-Wen Wang, Xue-Jun Li

**Affiliations:** 1 Department of Genetics and Developmental Biology, University of Connecticut Health Center, Farmington, CT 06030; 2 Department of Neuroscience, University of Connecticut Health Center, Farmington, CT 06030; 3 Pharmacokinetics, Dynamics, Metabolism-New Chemical Entities, Pfizer Worldwide Research and Development, Pfizer Inc., Groton, CT 06340; 4 Stem Cell Institute, University of Connecticut Health Center, Farmington, CT 06030

## Abstract

Nociceptive neurons play an essential role in pain sensation by transmitting painful stimuli to the central nervous system. However, investigations of nociceptive neuron biology have been hampered by the lack of accessibility of human nociceptive neurons. Here, we describe a system for efficiently guiding human embryonic stem cells into nociceptive neurons by first inducing these cells to the neural lineage. Subsequent addition of retinoic acid and BMP4 at specific time points and concentrations yielded a high population of neural crest progenitor cells (AP2α^+^, P75^+^), which further differentiated into nociceptive neurons (TRKA^+^, Nav1.7^+^, P2X3^+^). The overexpression of Neurogenin 1 (Neurog1) promoted the neurons to express genes related to sensory neurons (Peripherin, TrkA) and to further mature into TRPV1^+^ nociceptive neurons. Importantly, the overexpression of Neurog1 increased the response of these neurons to capsaicin stimulation, a hallmark of mature functional nociceptive neurons. Taken together, this study reveals the important role that Neurog1 plays in generating functional human nociceptive neurons.

Chronic pain is a debilitating condition, which directly affects about a fifth of the global population^1^. Unfortunately, current therapies are not sufficient for the majority of these patients as studies have shown that more than 50% of those treated do not experience a reprieve from their symptoms^2^. This is partially due to the lack of functional human nociceptive neurons available for researchers to study their biology and screen for therapeutic drugs against pain. Nociceptive neurons are on the front lines of pain sensation, as they are responsible for transmitting painful stimuli from the peripheral to the central nervous system^3^,^4^. Although nociceptive neurons are of the sensory lineage, they have major differences in function, morphology, and gene expression from mechanoreceptive and proprioceptive neurons^4^. Nociceptive neurons are typically tyrosine kinase receptor type 1 (TrkA) positive and have small cell bodies^5^. They can be subdivided into two characteristic groups; those which are myelinated (Aϐ) and fast conducting, and those which are unmyelinated (C-fibers) and slower conducting^6^. They can be further categorized by their status as either peptidergic or non-peptidergic^7^. In addition, the nociceptive neurons can express receptors such as transient receptor potential cation channel family V member 1 (TrpV1)^8^,^9^. TrpV1 positive cells are responsive to capsaicin as well as high temperatures and are widespread amongst the nociceptive neurons^8^,^10^. Since chronic pain affects a large portion of the population, it is critical that we develop a greater understanding of the development, maturation, and responsiveness of nociceptive neurons.

By using a chemically defined system and efficiently generating a robust population of neurons from human embryonic stem cells (hESCs)^11^,^12^,^13^, previous studies have shown that with slight but precise alterations to this system, many types of neurons such as spinal motor neurons^14^,^15^,^16^,^17^,^18^, midbrain dopaminergic neurons^19^,^20^,^21^, and neural retinal cells^22^,^23^ can be specified. Although some variations have been observed, differentiation protocols used for hESCs are also applicable to the other class of human pluripotent stem cells^24^,^25^,^26^, induced pluripotent stem cells (iPSCs)^27^,^28^. Thus, an efficient protocol to derive nociceptive neurons can be utilized to compare neurons derived from iPSCs of control patients and those of patients with pain disorders once they are established.

Neural crest precursors and sensory neurons, as shown by several recent studies^29^,^30^,^31^,^32^,^33^,^34^,^35^,^36^,^37^, have been generated from human pluripotent stem cells (hPSCs). However, how the specification of different human sensory neuron subtypes is regulated remains largely unclear, and the process by which a high population of functional capsaicin responsive nociceptive neurons can be efficiently generated eludes researchers. Here, we first differentiated hESCs into the neural lineage using our paradigm as previously described^11^,^38^. Based on evidence from developmental studies performed in other organisms, adaptations were made to this system in order to better recapitulate the spatial and temporal signals that the human nociceptive lineage would most likely be exposed to *in vivo*. As demonstrated in this study, we used developmental cues to differentiate nociceptive neurons from hESCs. These neurons express appropriate early and late stage markers and are functional showing a robust response to capsaicin stimulation. Moreover, we show that Neurogenin 1 (Neurog1) plays an important role in specifying nociceptive neurons from human pluripotent stem cells, but does not have an effect on other sensory neuron types.

## Results

### The specification of neural crest progenitors from hESCs

**Figure 1:**
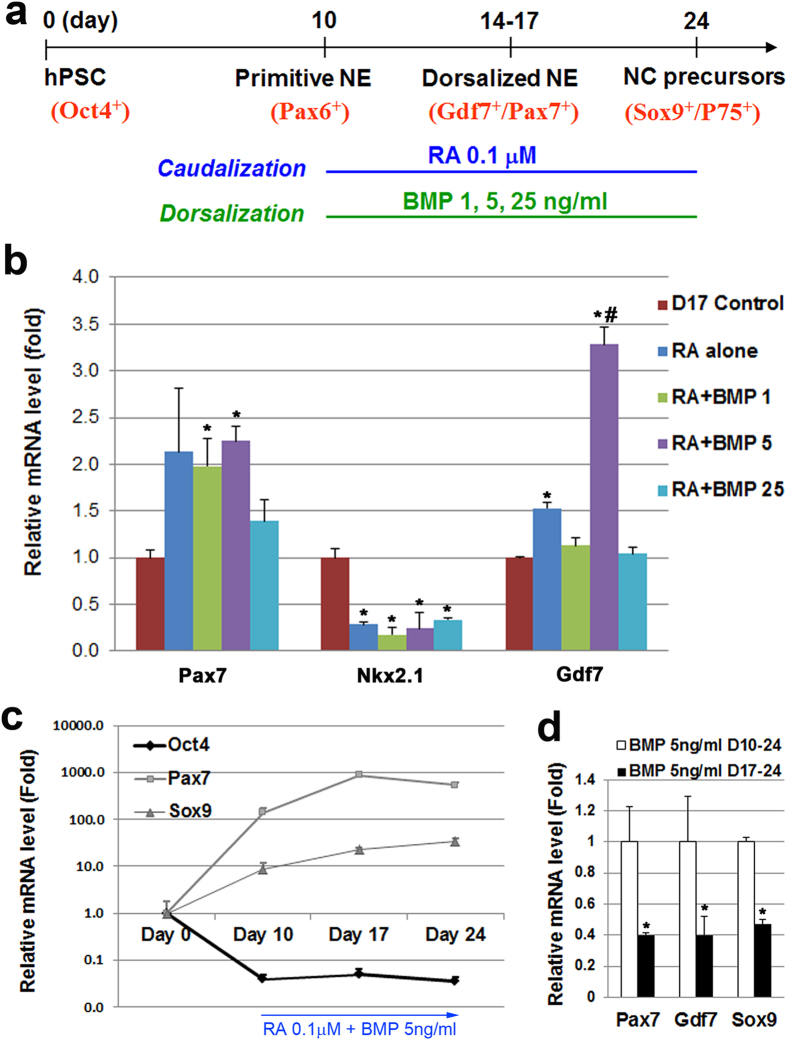
The effect of RA and BMP4 on cells in the neural differentiation paradigm. (**a**) A schematic of our timeline for the addition of RA and BMP4 to the cells. (**b**) RT-qPCR results showing the expression of dorsal and ventral markers at day 17 after differentiation when increasing concentrations of BMP4 were added during Days 10 to 17. (**c**) Quantitative PCR showing the temporal changes of pluripotent and neural crest-related genes during differentiation. (**d**) Quantitative PCR data comparing the expression of dorsal markers in cells at day 24 that were treated with BMP4 at early (D10-24) or late (D17–24) stages. Data are presented as mean ± SD, n = 3–4. *P < 0.05 versus Control group, ^#^*P* < 0.05 versus RA alone group.

The first step towards specifying nociceptive neurons was to generate neural crest progenitor cells from the neuroepithelia which are known to be the progenitors of the sensory lineage. The neural crest cells arise from the region between the dorsal part of neural and non-neural ectoderm^39^,^40^,^41^. It is also known that pain-related sensory neurons can come from the trunk (caudal) region. To influence the cells into this lineage, retinoic acid (RA) was added to induce caudalization. The concentration of 0.1 μM RA was selected because our lab previously established that this yields the highest population of caudalized cells without affecting their viability^16^,^17^. In terms of patterning signals for dorsalization, members of the transforming growth factor beta (TGFβ) family, especially bone morphogenetic proteins (BMPs), have been demonstrated to serve as dorsalizing signals in the early embryo^41^. Interestingly, BMP4 in particular has been shown to play an important role in initiating the formation of neural crest cells^42^,^43^. When added at specific time points in the differentiation process and at specific concentrations, BMP4 has been shown to increase the expression of peripheral sensory markers from mouse ESCs as well as Rhesus monkey ESCs^43^. Here, different concentrations of BMP4 (1 ng/ml, 5 ng/ml, 25 ng/ml) were added (Fig. 1a,b) to evaluate if BMP4 could promote the specification of neural crest lineage from hESCs. BMP4 and RA were applied to cultures at the primitive neuroepithelial stage (Day 10) (Fig. 1a). After 1 week of treatment, the expression of dorsal-ventral markers was examined at Day 17. Addition of BMP4 (5 ng/ml) and RA (0.1 μM) led to a significant increase in the expression of genes that are associated with the dorsal neural tube such as Gdf7 and Pax7, which is logical since it has been shown that the neural crest cells form in the more dorsal part of the neural tube^40^,^44^,^45^. In tandem, the expression of Nkx2.1 which is expressed more ventrally was significantly decreased in the presence of RA (Fig. 1b). Using the optimal conditions (BMP4 5 ng/ml plus RA 0.1 μM), we then examined the dynamic changes in gene expression during the neuronal differentiation from hESCs. There was a significant decrease in the expression of pluripotent marker (Oct4), but a dramatic increase in expression of the dorsal (Pax7) and neural crest marker (Sox9) during the differentiation from hESCs (Fig. 1c), suggesting the induction of the neural crest lineage.

Next, we examined the time window during which the application of BMP4 is critical for the generation of neural crest progenitors. Factors are often added after the mature neuroepithelial cells have formed. However, our lab has shown that cells are more responsive to inductive signals earlier on, when the primitive neuroepithelial cells have formed^13^,^16^. Thus, in the presence of RA (D10–24), we added BMP4 from the time of neural induction to the time of plating (D17–24 “late”) and compared the results with an earlier addition of BMP4 as described above (D10–24 “early”) at Day 24. The expression of dorsal (Pax7, Gdf7) and neural crest (Sox9) markers in the late BMP4 group was much lower than that of the early BMP4 group (Fig. 1d). Together, our data shows that early addition of BMP4 (from day 10) at a concentration of 5 ng/ml produced the optimal conditions, and thus was utilized in subsequent experiments.

### The differentiation of neural crest progenitors into mature sensory neurons

**Figure 2:**
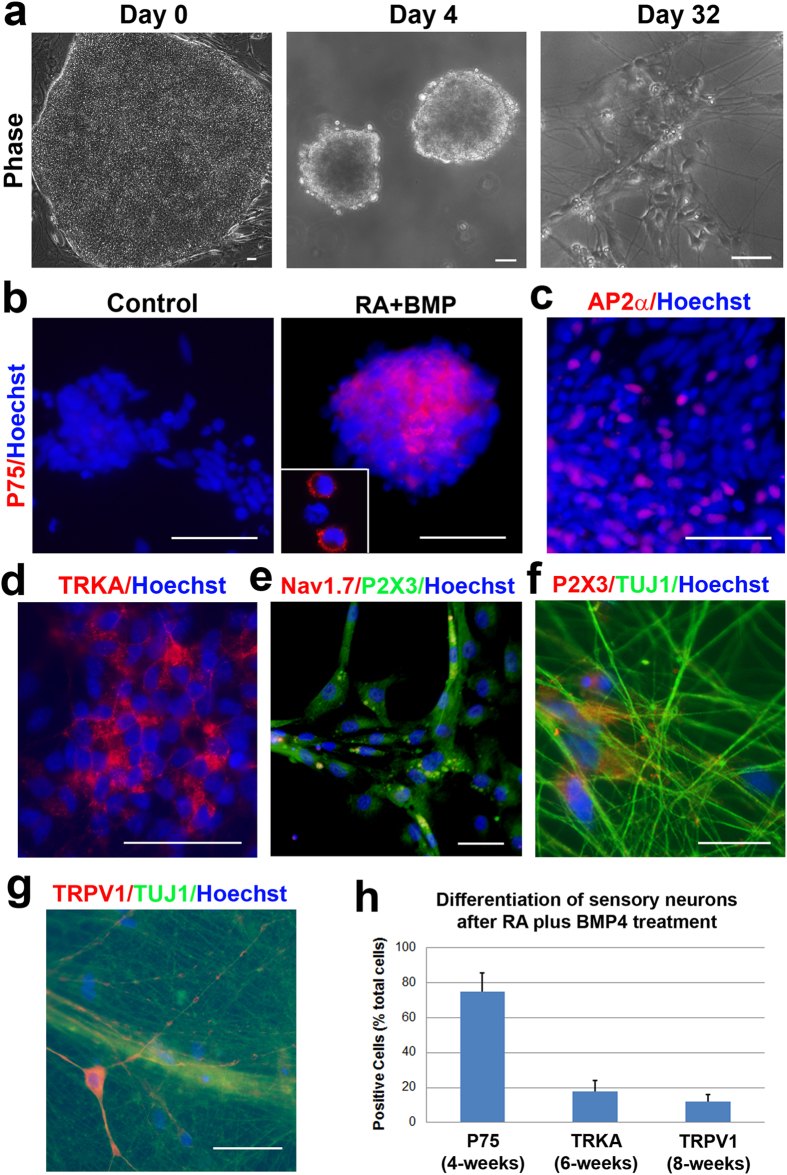
The generation of neural crest progenitors and nociceptive neurons from hESCs. (**a**) Phase-contrast images showing cells at multiple time points during differentiation into neural crest lineage. (**b**) Immunostaining showing the expression of P75 in Day 28 cultures from both control group (under basic condition; left panel) and the RA plus BMP group (treatment with RA and BMP4 from days 10–24; right panel). The insert in the right panel was taken at higher magnification (from a different field), indicating the membrane localization of P75. (**c**) At day 28 after differentiation, the neural crest progenitors were also positive for AP2α. (**d**) At 6 weeks after differentiation, immunostaining showed the expression of TrkA in neurons (6-weeks total) which had been originally treated with RA and BMP4. (**e**) In more mature neurons (8-weeks total), the expression of Nav1.7 and P2X3 were observed. (**f**) P2X3^ + ^cells were also positive for TUJ1, a neuronal marker, confirming that they were neurons. (**g,h**) At 8 weeks after differentiation from hESCs (8-weeks total), around 10% of cells became TRPV1^+^, which also double stained with a neuronal marker TUJ1. (**h**) Quantification of the differentiation of neural crest progenitors and sensory neurons. Data are presented as mean ± SD, n = 3. Bars, 50 (**a**–**e**,**g**) and 20 μm (**f**).

We then went on to evaluate the differentiation of the neural crest progenitors. When RA (0.1 μM) and BMP4 (5 ng/ml) were added from D10–24 and these progenitors were dissociated and plated onto poly-ornithine/laminin coated coverslips (Fig. 2a–c), this condition yielded a high population of neural crest progenitor cells, as indicated by the expression of P75 (Fig. 2b, right panel). This was in contrast to the cells under the control condition (with the same culture condition without RA and BMP4), where there were almost no P75^+^ cells (Fig. 2b, left panel). Importantly, these neural crest precursors (Day 28) were also positive for AP2α, another neural crest progenitor marker (Fig. 2c). When these cells were differentiated in culture for two more weeks (6-weeks total) in the presence of neurotrophic factors (nerve growth factor, NGF; glial cell line-derived neurotrophic factor, GDNF; growth differentiation factor 7, GDF-7; and insulin-like growth factor-1, IGF-1), a population of them stained positively for the nociceptive neuron marker TRKA (Fig. 2d). When these cells were allowed to mature for two additional weeks (8-weeks total), some of these neurons also stained positively for P2X3 and a sodium channel marker, Nav1.7 (Fig. 2e; Supplementary Fig. S1). The P2X3^+^ cells were also positive for βIII-tubulin (TUJ1), a neuronal marker, confirming that they were neurons (Fig. 2f). Around 10% of total cells expressed TRPV1^+^, a marker for nociceptive neurons (Fig. 2g,h). TRPV1^+^cells (8-weeks total) were double stained with the neuronal marker, TUJ1, indicating their neuronal identity (Fig. 2g). Thus, while we were able to induce approximately 75% of the cells to form neural crest progenitors, not all of those cells progressed to form mature nociceptive neurons (Fig. 2h).

### The Role of Neurog1 in specifying the nociceptive fate

**Figure 3:**
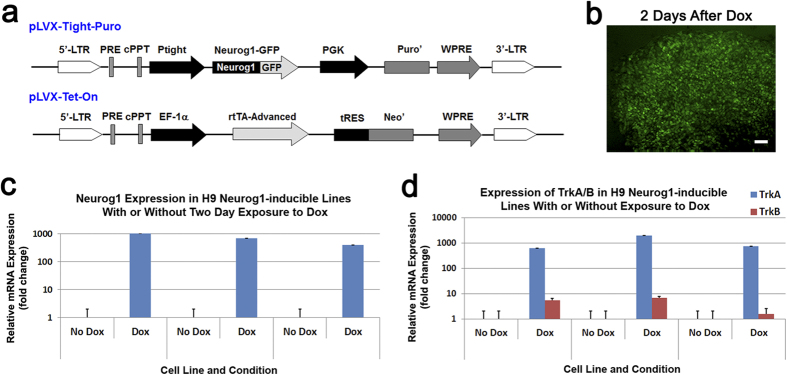
The generation and analysis of the Neurog1 inducible hESC lines. (**a**) Schematic map of the viral vectors utilized to make Neurog1 inducible lentiviruses which were transduced into hESCs. (**b**) After exposure to Doxycycline, the majority of the Neurog1 inducible cells became GFP positive, indicating that the system was functional. The Neurog1 inducible cells were selected and evaluated further. Bar, 50 μm. (**c**) As predicted, at 2 days after Doxycycline was added to the H9-Neurog1-inducible hESCs, there was a significant increase in Neurog1 expression. (**d**) Interestingly, even at the hESC stage, there was a substantial increase in TrkA expression but not TrkB expression when Neurog1 was overexpressed for 2 days. Data are presented as mean ± SD, n = 3.

In order to promote a higher percentage of neural crest progenitor cells into mature nociceptive neurons, we combined the application of morphogens and the genetic modification of Neurogenin 1 (Neurog1). It has been shown in other organisms such as mice^44^,^46^ and chicks^47^ that Neurog1 is an important factor in the cell fate decisions of sensory neurons. Importantly, Neurog1 has been shown to be necessary for the development of TRKA^+^ neurons, as they are absent in Neurog1 knockout mice^46^. Although less is known about its role in human development, we hypothesized that the overexpression of Neurog1 in our human system would promote the generation of nociceptive neurons. We first tested this by cloning Neurog1 cDNA into a lentiviral overexpression vector and generating lentiviruses to transduce neural crest progenitors. We observed an increase in the expression of TrkA when Neurog1 was overexpressed (Supplementary Fig. S2). To further elucidate this finding, we generated Neurog1 (tet-on, Doxycycline [Dox]) inducible H9 hESC lines using the pLVX-Tet-On Advanced inducible system (Fig. 3a,b). To evaluate the system at the hESC stage Dox was added (1 μg/ml) to the Neurog1 inducible hESCs. The expression of Neurog1, as indicated by the presence of GFP, could be induced after the Neurog1-inducible-H9 cells were treated with Dox (Fig. 3b). Since our medium does not have serum which may contain a trace amount of doxycycline (we used serum replacement for culturing hESCs), there was essentially no GFP expression before Dox treatment. In order to examine the effect of Neurog1 overexpression on hESCs, we then collected mRNA samples with and without a two day exposure of Dox and compared the gene expression (Fig. 3c,d). Treatment with Dox caused a robust increase in Neurog1 mRNA expression compared with the control (Fig. 3c). Since the basal expression levels of Neurog1 was low in hESCs, the fold change after the addition of Dox was quite high. Interestingly, when Neurog1 was overexpressed, there was a substantial increase in the TrkA expression (~1000 fold increase) but not the TrkB expression (~5 fold increase) (Fig. 3d) even at the hESC stage of development. At the ESC stage, the mRNA expression levels of TrkA and TrkB were extremely low and increased over a thousand times when cells differentiated into neural crest lineage at day 24. Therefore, the substantial increase in the expression of TrkA after the addition of Dox suggests that in humans Neurog1 may directly regulate and induce the expression of TrkA, a gene expressed by nociceptive neurons.

**Figure 4:**
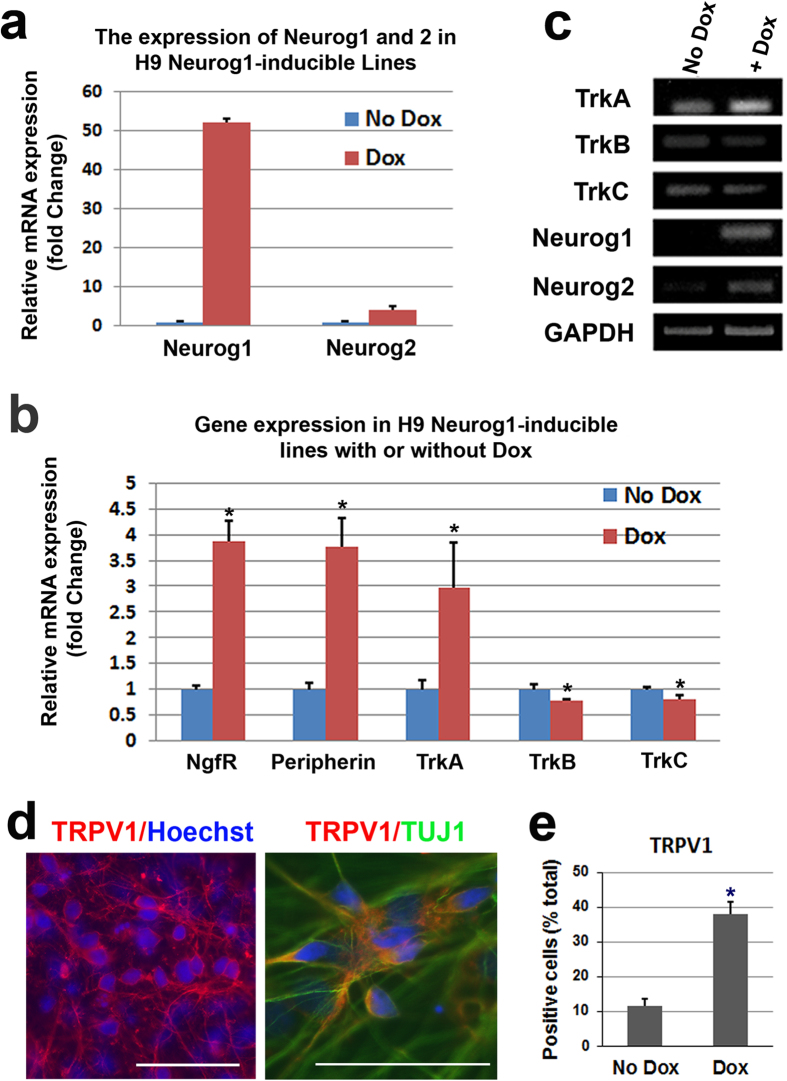
The overexpression of Neurog1 promotes the specification of the nociceptive lineage. (**a**) When Neurog1 was overexpressed from D17–24 (line 19) and evaluated at D24 there was a subsequent increase in the level of Neurog1. (**b**)These cells (line 19, D24) also showed an increase in the expression of sensory lineage gene expression such as NgfR and Periperhin. Importantly, when Neurog1 was overexpressed, there was a subsequent increase in TrkA which is necessary to form mature nociceptive cells, whereas there was little to no upregulation in the TrkB or TrkC expression. (**c**) Regular PCR of cells at Day 24 from the other line (line 18) showed a similar increase in the expression of TrkA, but not TrkB and TrkC, after the addition of Dox from D17–24. (**d,e**) After further culture (8-weeks), the Dox-treated cells (line 19) also had high expression of TRPV1, which was also positive for a neuronal marker TUJ1. Blue indicates Hoechst stained nuclei. Bar, 50 μm. Data are presented as mean ± SD, n = 3. *P < 0.05 versus No Dox group.

To examine the effect of Neurog1 overexpression on neural crest progenitors, the Neruog1 inducible hESCs were differentiated into neural crest progenitors as described above and Dox was added to the cells from the time point when they were mature neuroepithelial cells (Day 17) to the point at which the neurospheres were ready to be plated (Day 24) (Fig. 4a–c). The overexpression of Neurog1 by the addition of Dox (Fig. 4a) promoted the expression of sensory neuron-related genes NgfR and Peripherin (Fig. 4b) in the cultures (Day 24). Importantly, these Dox-treated cells showed a subsequent increase in TrkA expression which is critical for the development of nociceptive neurons (Fig. 4b,c). Also of importance, the overexpression of Neurog1 did not upregulate the expression of the TrkB or TrkC receptors which are imperative for the development of mechanoceptive and proprioceptive neurons (Fig. 4b,c). After further differentiation, cells in Dox-treated cultures expressed high levels of TRPV1, which also co-stained with a neuronal marker, TUJ1 (8-weeks, Fig. 4d). Notably, the proportion of TRPV1^+^neurons was much higher in Dox-treated group compared to that in control group (Fig. 4e). Together, these data reveal that Neurog1 promotes the generation of TRPV1^+^ nociceptive neurons.

### Evaluation of the function of nociceptive neurons

In order to further characterize the identity of our cell types as well as to evaluate whether or not our cells would be useful for testing new therapeutics, we performed calcium imaging to test the responsiveness of the cells using α,β-Methyleneadenosine-5′-triphosphate lithium and capsaicin. Other studies have shown that α,β-Methyleneadenosine-5′-triphosphate lithium induces the activation of the P2RX purinoceptor^48^. In both No Dox- and Dox-treated neural cultures (3.5 months), we applied α,β-Methyleneadenosine-5′-triphosphate lithium (30 μM) and compared the response of these cultures. Over 20% of the cells responded to the α,β-Methyleneadenosine-5′-triphosphate lithium (30 μM) in both No Dox- (7 out of 34 cells, 21%) and Dox-treated (4 out of 14 cells, 29%) groups, and the responses of the cells in these two groups were similar. It is known that capsaicin, the noxious factor in chili peppers is able to induce activation of nociceptive neurons that have the TrpV1 receptor^49^,^50^. The Dox-treated cultures had a much higher population of TRPV1^+^ neurons after differentiation (Fig. 4e). As expected based on results from our staining, there was a dramatic increase in the response to capsaicin (1 μM) from the cells that were exposed to Neurog1 (+Dox, 45 out of 71 [63%] cells responded) relative to those which were not exposed to Neurog1 (no Dox, 4 out of 29 [14%] cells responded).

**Figure 5:**
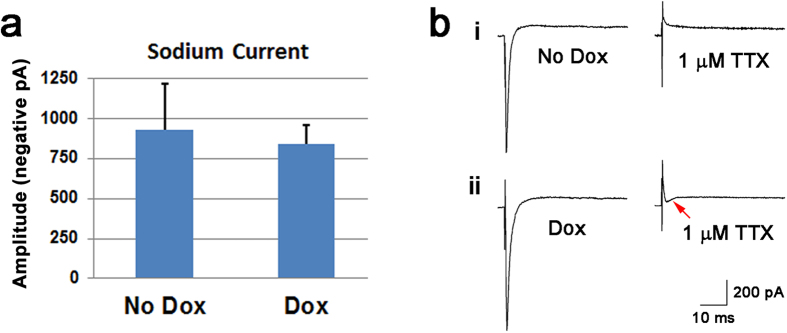
Sodium currents in hESC-derived neurons. (**a**) Sodium currents were observed by patch clamp in 8-week neurons. There were no differences between the amplitudes in the No Dox- and Dox-treated groups. Data are presented as mean ± SE, n = 5–7. (**b**) After applying TTX (1 μM), the sodium current was totally blocked in a control neuron (**i**); however, a small TTX-resistant current (indicated by an arrow) was observed in the Dox-treated neuron (**ii**).

One feature of functional nociceptive neurons is that they could have both tetrodotoxin (TTX)-sensitive and TTX-resistant sodium channels^51^,^52^,^53^,^54^,^55^. Using voltage clamp recordings, we examined the sodium currents in both no Dox- and Dox-treated neurons (Fig. 5a). Sodium currents were observed and there were no significant differences in the amplitude between the no Dox- and Dox-treated neurons (Fig. 5a). Interestingly, we observed both TTX-sensitive and TTX-resistant sodium channels in the Dox-treated neurons (Fig. 5b), further supporting the functionality of the nociceptive neurons in our cultures. Taken together, these results suggest that when human cells are exposed to Neurog1 during these critical time windows of development, they are pushed toward the nociceptive lineage.

## Discussion

Chronic pain is both debilitating and extremely common, but current treatments are unfortunately ineffective for the majority of sufferers^1^. This is partially due to the lack of *in vitro* human systems on which to test new treatments, and a solid knowledge base regarding how these neurons develop in humans. Nociceptive neurons are poorly understood, but they are at the forefront of the pain pathway relaying information regarding noxious stimuli from the periphery to the central nervous system. In this study, we describe the generation of functional nociceptive neurons from human embryonic stem cells using a chemically defined and highly reproducible system which mimics developmental principles. The addition of specific morphogens (RA, BMP4) during a critical time window yielded a high population of neural crest (AP2α^+^, P75^+^) cells. We showed that a high population of these cells expressed genes indicative of the sensory lineage. More importantly, we demonstrated the critical role of Neurog1 in specifying nociceptive neurons. With the combination of morphogens and Neruog1 overexpression, nociceptive neurons can be efficiently generated from human pluripotent stem cells.

Neurogenin 1, a member of the neurogenin family, plays an important role in neuronal differentiation. A recent study showed that overexpression of Neurog1 and Neurog2 could derive functional neurons from human iPSCs at an accelerated pace^30^. The important role of neurogenins in human sensory neuron differentiation was supported by two studies in which human fibroblast cells were directly converted into sensory neurons with transcription factors including neurogenins^56^,^57^. The overexpression of Brn3a and Neurog1 or Neurog2 yielded a population of sensory neurons from human fibroblasts which could not be achieved by the overexpression of any of the genes individually^56^. Interestingly, the co-expression of Brn3a and Neurog1, as compared to Brn3a and Neurog2, led to a much higher increase in the TrkA expression at around 7–17 days post reprogramming. In this study, by direct differentiation of hESCs into sensory neurons, we found that the overexpression of Neurog1 alone could promote the specification of TrkA^+^, but not TrkB^+^ or TrkC^+^, sensory neurons from hESCs. Our findings reveal the critical role of Neurog1 for the differentiation of TrKA^+^ human nociceptive neurons and are consistent with an *in vivo* study performed in mice in which the knockout of Neurog1 led to the complete loss of TrkA^+^ sensory neurons in the DRG^46^. In contrast, only a subset of TrkB^+^ and TrkC^+^ neurons (~25%) were lost, while all were lost in Neurog1- and Neurog2- double knockout mice^46^. Our data also reveal that the concentration of BMP4 is important for differentiating hESCs into sensory lineage, a finding that coincides with a previous study using mouse and monkey ESCs^43^.

The neural differentiation method that we used in this study began with the formation of embryoid bodies (EBs, also known as “ESC aggregates”), a system commonly utilized for neural differentiation. Although this system (via “EB-formation”) takes a longer time to generate neuroepithelial cells (2-weeks versus 1-week using monolayer differentiation), the rationale for suspending the cells as aggregates is to initiate spontaneous differentiation in order to mimic early development (see review and papers from Dr. Su-Chun Zhang’s group for a detailed discussion on this method^11^,^12^,^58^). Similar to what has been previously described^16^,^59^, primitive NE cells were generated at day 10 after differentiation. With the treatment of RA and BMP4, neural crest progenitors were generated at around day 24 of differentiation, and these progenitors were further differentiated into sensory neurons after 6-weeks of differentiation. A major advantage of this stepwise system is that one can easily identify the different stages and can tease apart the roles of different factors at distinct stages of differentiation. After establishing the Dox-inducible-Neurog1 H9 hESCs, we investigated the role of Neurog1 on generating nociceptive neurons in this study. Our data showed that the overexpression of Neurog1 (Day 17–24) could explicitly promote the specification of TrkA^+^ sensory neurons from hESCs. More importantly, the overexpression of Neurog1 promoted the generation of TRPV1^+^ nociceptive neurons (over 35% in 8-week cultures). This is further supported by the increased response to capsaicin stimulation in Dox-treated cells (63% Dox-treated-cells versus 14% no-Dox-treated-cells) in long-term cultures (3.5 months). Some, but not all Dox-treated-cells showed an enhanced response to capsaicin as indicated by the ratiometric fluorescence signal (the peak F/F0 in Dox-treated cells is 0.50 versus 0.11 in No-Dox-treated cells). Since cells detach easily in long-term cultures, it will be valuable to investigate more cells at different stages of maturation in the future. Nevertheless, the hESC-derived nociceptive neurons are functional, as revealed by their response to capsaicin stimulation (comparable to DRG neurons^60^) and the possession of TTX-resistant and sensitive sodium currents^51^,^52^,^53^,^54^. Therefore, this study lays the groundwork for yielding the appropriate sensory precursors, and provides a unique paradigm to generate functional nociceptive neurons from hPSCs based on developmental principles. Interestingly, the overexpression of Neurog1 at the ESC stage induced a substantial increase in the expression of TrkA in these stem cells which innately have an extremely low expression of TrkA. These data suggest that TrkA may be a direct target of Neurog1, which will be interesting to investigate in the future. Furthermore, considering the distinct roles of Neurog1 and Neurog2 in sensory neuron development^44^,^46^, it is possible to specify different sensory neuronal subtypes by differentially regulating these genes.

Learning more about how human cells respond to various conditions and novel therapeutics can lead to tremendous breakthroughs in the treatment of pain as well as developmental disorders. The generation of nociceptive neurons derived from human stem cells opens up many doors as it provides a renewable, consistent, and unlimited supply to study for experimentation from mechanistic, drug-discovery, and clinical perspectives. The investigation of the effects of chemotherapeutic drugs on these cells to evaluate their role in neuropathic pain in humans will be intriguing. In addition, generating inducible hESCs that could express or knock out critical genes in the pain pathway will be useful. Our well-characterized chemically defined system also provides a platform for generating other similar cell types that we hope will yield additional advancements. Now that hiPSC technology is feasible^24^,^27^,^28^, we can use these strategies to differentiate iPSCs from individuals with genetic mutations in genes such as SCN9A^61^ in order to understand the potential differences in their nociceptive neurons, and how to better treat patients.

## Methods

### hESC cultures

H9 cells were cultured on a layer of mouse embryonic fibroblast cells (MEFs) in hESC medium consisting of DMEM/F12 (Life Technologies, Carlsbad, MA) with 20% KnockOut Serum Replacer (Life Technologies), 0.1 mM Non-essential Amino Acids (NEAA, Life Technologies), 1 mM L-glutamine (Life Technologies), and 0.1 mM β-mercaptoethanol (Sigma-Aldrich, St. Louis, MO) and then supplemented with 4 ng/ml bFGF (Life Technologies).

### Differentiation of hESCs

The standard protocol for differentiating hESCs into neural lineage has been previously published^11^,^38^ and further details including a video are available^38^. In brief, colonies were detached from feeder cells (at day 0) and suspended in hESC medium (same as described in the above section, without bFGF) for 4 days. The ESC aggregates (also called “embryoid bodies”, EBs) were then cultured in a neural induction medium (NIM) with DMEM/F12, 1% N2 supplement (Life Technologies), 1% NEAA, and 2 μg/ml heparin (Sigma-Aldrich) for two days. After adherence to a plastic surface on day 7 or 8, primitive neuroepithelial (NE) cells were observed around day 10 (Supplementary Fig. S3). At day 17 the neuroepithelium was isolated by gently triturating the whole colonies using a 1ml pipette. These cells were then suspended in neural induction medium with 2% B27 (Life Technologies) and 1 μM cAMP. The cells were then transferred to a non-tissue culture treated T25 flask with 10 ml of NIM. At day 24 the neurospheres were dissociated into small clusters using Accutase (Innovative Cell Technologies, San Diego, CA) and were plated onto coverslips coated with polyornithine and laminin in differentiation media (NDM) with 50/50 Neurobasal and DMEM/F12, 1% B27, 1% N2, 1 μM cAMP (Sigma-Aldrich), 200 μM ascorbic acid (Sigma-Aldrich), and 10 ng/ml NGF, GDNF, IGF-1, and GDF-7 (PeproTech, Inc, Rocky Hill, NJ). In the experimental groups for generating sensory neurons (Supplementary Fig. S3), retinoic acid (0.1 μM, Sigma-Aldrich) and BMP4 (1, 5 or 25 ng/ml) were added as described in the results. For each of these steps, the medium was changed every other day. Once the cells were in NIM, half of the medium was changed every other day.

### Lentiviral production and transduction

The lentivirus was produced using HEK293-FT cells by transfecting them via the calcium phosphate method with 7.5 μg of psPAX2 (Addgene, Cambridge, MA), 10 μg of the lentiviral transfection vector, and 5 μg of the VSV-G envelope protein pMD2.0G (Addgene). Seventeen hours after transfection, the medium was replaced. After 36 hours, the cell culture medium containing the viral particles was collected and filtered through a 0.45 μm filter (Millipore, Billerica, MA). The viral particles were further concentrated by ultracentrifugation (SW 28 rotor, Beckman Coulter, Inc., Brea, CA) at 50,000 g for 2 hours at 16 °C. The pellets were resuspended in hESC medium. For the transduction of ESCs, the cells from one well of a 6 well plate (~1 million) were passaged using standard technique and pelleted via centrifugation. The cell pellets were then incubated with 100 μl of concentrated virus at 37 °C for 30 minutes. During the incubation period, the cells were gently mixed every 10 minutes. The cells and the virus were then transferred to a 6 well plate containing standard medium and a MEF feeder layer overnight. The medium was then changed the next day.

### Generation of Neurog1 inducible lines

To generate the inducible hESC lines, the LentiX-Tet-On Advanced Inducible Expression System (Clonetech, Mountain View, CA) was utilized. The CMV promoter in the pLVX-Tet-On Advanced vector was exchanged for EF1α (Addgene). To change the promotor, the pLVX-Tet-On Advanced vector was first digested using the two enzymes which flanked the CMV promoter (BamHI and ClaI). This was then ligated with the EF1α promoter which was PCR amplified using primers with BamHI and ClaI sequences. This is a similar method to what we have previously described^62^. The Neurog1 sequence was added into the multiple cloning site of the pLVX-Tight-Puro vector in order to generate cells, which overexpressed this gene when exposed to Dox. Lentivirus was made to express each of these. Each vector had a drug resistance gene (neomycin or puromycin) and after the cells were exposed to the lentivirus, they were exposed to the appropriate drug (G418, 50 μg/ml; Puromycin, 1 μg/ml; Gemini, West Sacramento, CA) for 10 days until only those which were resistant remained.

### RT-PCR and qPCR

Total RNA was extracted from the cells using TRIzol (Life Technologies). The SuperScript kit (Life Technologies) was utilized to synthesize cDNA. PCR was used to assess gene expression with primers for specific genes. Quantitative PCR (qPCR) was performed with the appropriate SYBR Green gene expression assay in a 20μl mixture containing cDNA, primers and 1 × iQ SYBR Green Supermix (Bio-Rad, Hercules, CA). Standard curves were measured by each set of primers to confirm that only one amplicon was generated at the same efficiency as glyceraldehyde 3-phosphate dehydrogenase (Gapdh), a house keeping gene. Expression level of the mRNA was calculated using the comparative ΔC_T_ method.

### Immunocytochemistry and quantification

The coverslips were fixed using 4% paraformaldehyde (Sigma-Aldrich)^16^. Next, they were rinsed with PBS (Life Sciences), and were incubated with 0.2% Triton X-100 (Sigma-Aldrich) for 10 minutes and 10% normal donkey serum (Jackson ImmunoResearch Laboratories, Inc. West Grove, PA) for 1h before overnight incubation with primary antibodies in PBS containing 5% donkey serum at 4 °C. Antigen-antibody reactions were developed by the appropriate fluorescein-conjugated secondary antibodies (Invitrogen and Jackson ImmunoResearch Laboratories, Inc.). Nuclei were stained with Hoechst (Invitrogen). Primary antibodies used in this study included βIII-tubulin (Tuj1, 1:1000, mouse IgG, Developmental Studies Hybridoma Bank [DSHB]), TrpV1 (Neuromics, guinea pig IgG, 1:1000), Nav1.7 (Neuromics, mIgG, 1:750), P75 (Advanced Targeting Systems, mIgG, 1:200), P2X3 (Millipore, guinea pig IgG, 1:1000), TrkA (R&D, goat IgG, 1:100), and AP2α (DSHB, mIgG, 1:200). Images were collected using a Zeiss camera mounted onto Zeiss Axiovert 200M fluorescence microscope or Zeiss LSM 510 Meta confocal microscope (Carl Zeiss Inc., Thornwood, NY.). To confirm the specificity of the staining, negative controls without primary antibodies were included in which no positive fluorescence signals were observed.

The population of P75, TrkA, or TrpV1-expressing neurons among total differentiated cells (Hoechst labeled) was counted as described previously^13^,^16^. Briefly, a trained person who was blinded to the experimental conditions used a Zeiss fluorescence microscope to image 4 fields on each coverslip. These 4 images were taken under 40X magnification in areas where individual neurons could be seen. Next, the labeled cells in these images were manually counted using Metamorph software. These images were taken using the same exposure time at which the positive and negative cells could be easily identified. For each coverslip, the selected fields had between 108–259 cells. The counting and imaging was performed by a person who was blinded to the experiments to ensure that the same criteria are used. For each group, 3 coverslips were counted. The statistical significance of mean differences between No Dox- and Dox-treated groups was analyzed using two-tailed Student’s t-test.

### Calcium dye

The calcium dye Fluo-4 AM (Life Technologies) was prepared at a 5mM concentration (10 μl DMSO, 50 μg dye). This was then placed into 100 μl of Powerload (Life Technologies), and 10ml of calcium-free PBS. Any unused portions were immediately aliquoted, wrapped in parafilm, and frozen at −20 °C. The neurons were incubated in the Fluor-4 solution for 30 minutes at 37 °C, for 30 minutes at room temperature, and then rinsed with PBS. Since cells do not remain attached very well in long-term cultures, some cells were lost after washing and staining with the dye. The remaining cells were subjected to the calcium imaging and analysis.

### Calcium imaging and evaluation

When performing the calcium imaging, we observed the morphology of the cells under the microscope and examined those with a typical neuronal morphology. For each coverslip of neurons, the flux of calcium was recorded for 3 minutes with a 15 ms exposure time and a 100ms delay between the exposures. The fluorescence was recorded both before and during exposure to capsaicin (1 μM, Sigma-Aldrich), or α,β-Methyleneadenosine-5′-triphosphate lithium (30 μM, Sigma-Aldrich). Fluorescent images were obtained with a 40X objective (aperture 1.3) using a Nikon TE2000S inverted epifluorescence microscope with an Electron-multiplying CCD camera (iXonEM + 885, Andor Technology, Belfast, Northern Ireland) which was equipped with a FITC filter (#59222, Chroma Technology Corp., Bellows Falls, VT, USA), as well as a light source (Lambda XL, Sutter Instrument, Novato, CA, USA). To measure the change in fluorescence intensity, a line was circled around the perimeter of the cell and specified as a region of interest (ROI). The signal was evaluated on a pixel-by-pixel basis evaluating the contrast (F/F0) in fluorescence between the baseline and treatment and determined for each cell. Responsive cells were defined with a change in F/F0 (the ratiometric fluorescence signal) larger than 0.25. The evaluation was done using the NIS-Elements software V3.22.11 (Nikon, Tokyo, Japan) and then a custom-designed MATLAB software (The Mathworks, Inc., Natick, MA, USA).

### Electrophysiology

Coverslips were placed in a bath solution including the following (in mM): 140 NaCl, 4.7 KCl, 2.5 CaCl2, 1.2 MgCl2, 10 HEPES and 10 glucose; pH was adjusted to 7.4 with NaOH. The intracellular (pipette) solution for voltage-clamp contained (mM): 100 CsF, 45 CsCl, 10 NaCl, 1 MgCl2, 10 HEPES, and 5 EGTA; pH was adjusted to 7.3 with CsOH. Whole cell voltage-clamp was obtained using a MultiClamp 700B amplifier (Molecular Devices, Sunnyvale, CA). Sodium currents were elicited by current injections from a holding potential of −80 mV to −10 mV for 500 ms and recorded for total 800 ms. Tetrodotoxin (TTX; Sigma) was applied to the cells at a final concentration of 1 μm. All data were analyzed with pClamp 10.0 (Molecular Devices) software.

### Statistical analysis

The statistical significance in mean values among multiple sample groups was analyzed with Tukey’s range test after ANOVA. Two-tailed Student’s t-test was used to examine the statistical significance between two sample groups. The significance level was defined as *P* < 0.05, and significance tests were conducted using SAS 9.1 (SAS Institute).

## Additional Information

**How to cite this article**: Boisvert, E. M. *et al.* The Specification and Maturation of Nociceptive Neurons from Human Embryonic Stem Cells. *Sci. Rep.* **5**, 16821; doi: 10.1038/srep16821 (2015).

## Supplementary Material

Supplementary Information
