# Supplementary material for: The Specification and Maturation of Nociceptive Neurons from Human Embryonic Stem Cells
Source: Sci Rep. 2015 Nov 19;5:16821. doi: 10.1038/srep16821 (PMC4652175; doi:10.1038/srep16821)
Supplement: Supplementary Information [file srep16821-s1.pdf]

SUPPLEMENTARY INFORMATION:

The Specification and Maturation of Nociceptive Neurons from Human Embryonic Stem Cells

Erin M. Boisvert[1,2], Sandra J. Engle[3], Shawn E. Hallowell[3], Ping Liu[2], Zhao-Wen Wang[2], Xue-Jun Li[2,4]*

[1]Department of Genetics and Developmental Biology, University of Connecticut Health Center, Farmington, CT 06030. [2]Department of Neuroscience, University of Connecticut Health Center, Farmington, CT 06030. [3]Pharmacokinetics, Dynamics, Metabolism-New Chemical Entities, Pfizer Worldwide Research and Development, Pfizer Inc., Groton, CT 06340. [4]Stem Cell Institute, University of Connecticut Health Center, Farmington, CT 06030.

Contents:

Supplementary Figure S1
Supplementary Figure S2
Supplementary Figure S3

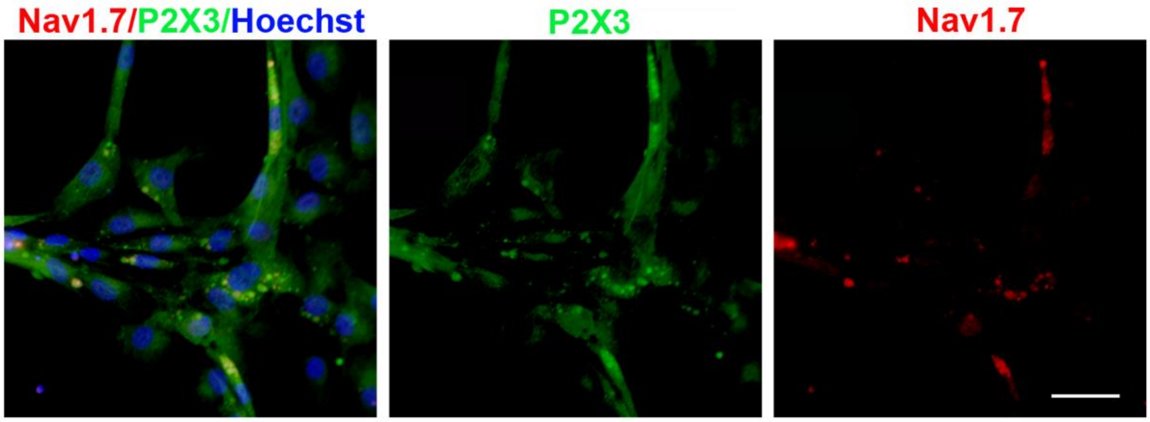

Supplementary Figure S1. Immunostaining showing the expression of P2X3 and Nav1.7 by sensory neuron cultures 8-weeks after differentiation from hESCs. Blue indicates Hoechst stained nuclei. Scale bar, 50 μm.

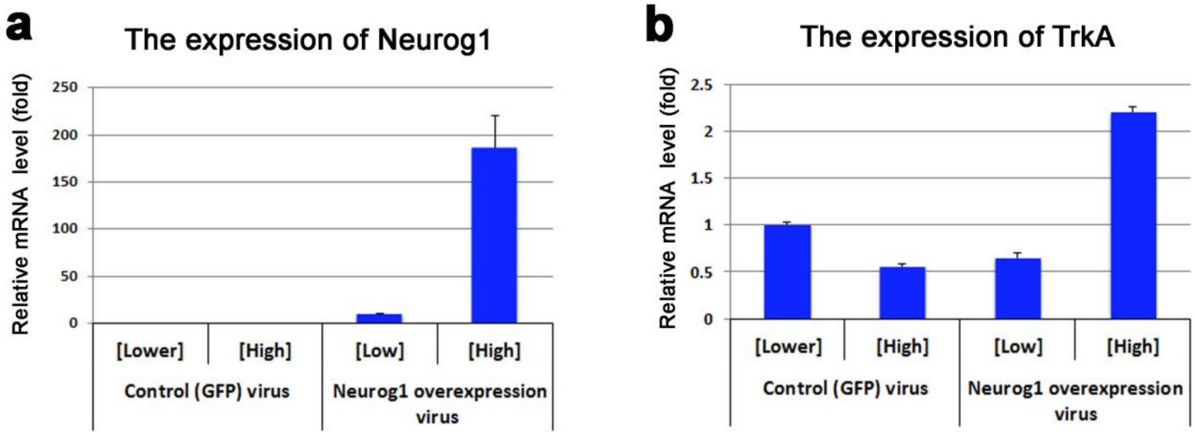

Supplementary Figure S2. The expression of Neurog1 and TrkA when the cells were exposed to either control (GFP) or Neurog1 overexpression lentivirus. The addition of control (GFP) overexpression virus at lower (3 µL) or higher (10 µL) concentrations had little effect (**a,b**). When Neurog1 overexpression virus was added at a higher concentration, the expression of Neurog1 increased (**a**), as did the expression of TrkA (**b**).

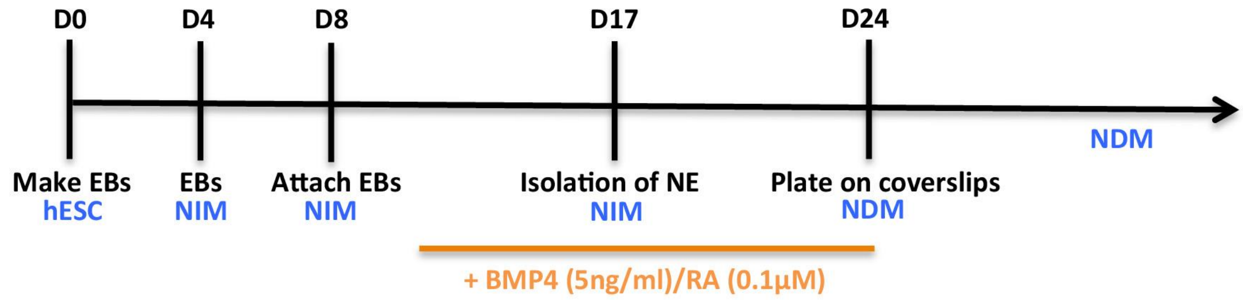

Supplementary Figure S3. A schematic procedure of neural differentiation from hESCs.
